# Space–time shape uncertainties in the forward and inverse problem of electrocardiography

**DOI:** 10.1002/cnm.3522

**Published:** 2021-09-08

**Authors:** Lia Gander, Rolf Krause, Michael Multerer, Simone Pezzuto

**Affiliations:** ^1^ Center for Computational Medicine in Cardiology Euler Institute, Università della Svizzera italiana Lugano Switzerland

**Keywords:** $H^{1/2}$ regularisation, boundary integral formulation, inverse problem of electrocardiography, quasi‐Monte Carlo method, space‐time shape uncertainty, sparse quadrature

## Abstract

In electrocardiography, the “classic” inverse problem is the reconstruction of electric potentials at a surface enclosing the heart from remote recordings at the body surface and an accurate description of the anatomy. The latter being affected by noise and obtained with limited resolution due to clinical constraints, a possibly large uncertainty may be perpetuated in the inverse reconstruction. The purpose of this work is to study the effect of shape uncertainty on the forward and the inverse problem of electrocardiography. To this aim, the problem is first recast into a boundary integral formulation and then discretised with a collocation method to achieve high convergence rates and a fast time to solution. The shape uncertainty of the domain is represented by a random deformation field defined on a reference configuration. We propose a periodic‐in‐time covariance kernel for the random field and approximate the Karhunen–Loève expansion using low‐rank techniques for fast sampling. The space–time uncertainty in the expected potential and its variance is evaluated with an anisotropic sparse quadrature approach and validated by a quasi‐Monte Carlo method. We present several numerical experiments on a simplified but physiologically grounded two‐dimensional geometry to illustrate the validity of the approach. The tested parametric dimension ranged from 100 up to 600. For the forward problem, the sparse quadrature is very effective. In the inverse problem, the sparse quadrature and the quasi‐Monte Carlo method perform as expected, except for the total variation regularisation, where convergence is limited by lack of regularity. We finally investigate an $H^{1/2}$ regularisation, which naturally stems from the boundary integral formulation, and compare it to more classical approaches.

## Graphical abstract

The solution to the forward and inverse problems of electrocardiography requires a description of the torso anatomy. The latter varies over time and is source of uncertainty, since it is obtained from images with limited resolution. We study the effect of the space–time shape uncertainty on the forward and inverse solutions. The uncertainty is represented by a random deformation field with high‐dimensional Karhunen–Loève expansion. Therefore, we employ the anisotropic sparse quadrature to estimate our quantities of interest.

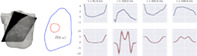

## 1 INTRODUCTION

Electrocardiographic recordings at the body surface, such as the standard 12‐lead electrocardiogram (ECG), are a direct consequence of the electric activity of the heart. The spatial resolution of such recordings depends on the number of electrodes placed on the chest, ranging from no more than 10 of the standard ECG to hundreds of electrodes composing high‐density body surface potential maps (BSPMs). Along with an accurate description of the torso anatomy, BSPMs are sufficiently informative to enable a non‐invasive characterisation of cardiac electrophysiology, termed ECG imaging.^1^ ECG imaging technologies have been extensively validated in experimental, animal and, more recently, clinical settings, with promising results.^2^

The ECG imaging problem can mathematically be formulated as an inverse problem. The most classical formulation of it is associated with a potential‐based forward problem, which amounts to determining the BSPM on the chest from the electric potential at a surface enclosing the heart, for example, the epicardium. The inverse problem of electrocardiography consists therefore in recovering the epicardial potential from the BSPM.^3^ As typical for inverse problems, however, the ECG imaging problem is severely ill‐posed in the sense of Hadamard, since arbitrarily small perturbations of the BSPM, such as noise, may yield large variations in the epicardial potential. It thus requires regularisation to penalise non‐physical solutions and a strategy to optimally select the associated regularisation parameter. For the ECG inverse problem, several kinds of regularisations have been proposed over the last three decades, see Reference 4 for a review. The standard Tikhonov regularisations of zeroth, first or second order^3^, ^5^ are easy to implement thanks to the closed‐form solution of the quadratic inverse problem. Closely related to the Tikhonov regularisation is the generalised truncated singular value decomposition, in which small singular values of the forward operator are filtered out.^6^ The inverse problem can also be interpreted from a Bayesian perspective.^7^ In this case, the maximum a posteriori estimator, which matches the classical Tikhonov solution under the usual hypothesis of a standard Gaussian prior distribution, offers more flexibility in embodying prior knowledge in the inverse problem, for example, from a training data set. Non‐smooth regularisations such as total variation (TV) are a valid alternative to quadratic approaches in the presence of sharp gradients in the reconstructed data,^8^ but lead to a significantly more difficult solution of the inverse problem. Approximated or smoothed versions of TV are therefore popular.^4^ More recently, physiology‐based and spatio‐temporal regularisation approaches have also been considered.^9^, ^10^

The potential‐based forward problem is particularly attractive when the torso is assumed as a homogeneous electric conductor. Then, the forward problem can be conveniently recast into an integral formulation involving only the boundaries of the torso, that is, the epicardium and the chest, with no need of solving the problem in the full three‐dimensional (3D) domain.^11^, ^12^ The numerical treatment of the boundary formulation is however less practical than a standard finite element approach in 3D, as it requires special care in the treatment of singular boundary integrals on piecewise smooth surfaces, like triangulated surfaces obtained from the segmentation of cardiac images. Acquired with given clinical constraints, imaging data are typically of limited resolution and noisy. Therefore, the segmentation of selected cavities is challenging and certainly subject to uncertainty. In the case of the heart, segmentation is made even more difficult by the movement of the organ. As a consequence, the resulting segmentation is subject to time‐dependent shape uncertainty. This uncertainty in the anatomy propagates through the solution of the inverse problem, resulting in a reconstruction also affected by uncertainty.

In spite of its acknowledged importance,^13^ uncertainty quantification in the context of cardiac modelling has emerged only very recently, see for example References 14, 15, 16, 17. In electrocardiography, the most relevant uncertainty to account for is in the body surface electric recordings. Within a Bayesian framework, the inverse problem maps a prior distribution of the pericardial potentials into a posterior distribution, which also accounts for noise in the input data through the likelihood.^7^ Model uncertainty has been considered as well. The electric conductivity of the torso is highly heterogeneous, for example, lungs, blood masses, interstitial tissue, muscles and bones significantly differ in terms of conduction, and uncertain, which may influence the reconstruction as well.^18^, ^19^ In the present context, shape uncertainty has however received very limited attention thus far, despite preliminary studies showed a non‐negligible impact on the inverse reconstruction.^20^, ^21^

From the mathematical perspective, sophisticated tools and theory for the treatment of shape uncertainties are already available. Besides the fictitious domain approach considered in Reference 22, one typically distinguishes two approaches to deal with shape uncertainties: the perturbation method, see Reference 23 and the references therein, which is suitable to treat small perturbations of the nominal shape and the domain mapping method, see References 24, 25, 26, 27. Here, we focus on the more flexible domain mapping approach. Then, the computation of quantities of interest, such as expectation and variance of the potential, gives rise to high dimensional quadrature problems for the random parameters. Given sufficient parametric regularity, sophisticated sparse quadrature and quasi‐Monte Carlo methods, see for example References 28, 29, 30, 31, 32, can be applied. If such regularity is not present, one has to resort to the only slowly converging, Monte Carlo method.

This work aims at evaluating the impact of space–time uncertainty on the forward and inverse problems of electrocardiography. To model the shape uncertainty, we consider a space–time reference geometry and a random deformation field, that we represent by its Karhunen–Loève expansion. The covariance of the random deformation field depends on both space and time and accounts for the periodicity of the motion of the heart. Furthermore, we formulate the forward problem as a boundary integral equation by assuming a constant torso conductivity. We refer to Reference 12 for all the details of this approach and in particular for the applied discretisation. The boundary integral formulation is particularly suitable for our purpose, because both input data and observations are confined to a surface, these are the pericardium and the chest, respectively. In order to accelerate the computation of quantities of interest, we discretise the spatial problem with a rapidly converging collocation method, achieving high accuracy already with a relatively coarse mesh, while the deformation field is approximated by an efficient low‐rank technique.

In order to assess the effect of the shape uncertainty, we estimate the expectation and the variance of the chest potential (forward problem) and the pericardial potential (inverse problem) using the anisotropic sparse quadrature method from Reference 28. This approach is validated against the quasi‐Monte Carlo method based on Halton points, see Reference 32. The inverse problem, known to be severely ill‐posed, needs an adequate regularisation. We employ classic regularisations proposed specifically for our problem of interest, such as zero order Tikhonov, the first‐order Tikhonov and TV.^4^ The implication of the choice of the regularisation on the convergence rate of the sparse quadrature is also analysed. Finally, we explore an $H^{1/2}$ regularisation, see References 33, a natural option stemming from the boundary integral formulation of the problem itself.

The paper is organised as follows. In section 2, we introduce the mathematical description of the problem at hand. Section 3 provides the corresponding boundary integral formulation and the discretisation of the involved boundary integral operators. The different regularisation methods for the inverse problem are presented in section 4. Section 5, dedicated to modelling shape uncertainty, is the cornerstone of the present work. Finally, section 6 is devoted to the numerical assessment of a two‐dimensional, Cine Magnetic Resonance Imaging (MRI)‐derived geometry. Herein, we validate the sparse quadrature and we quantify the effect of the shape uncertainty on the forward and inverse problems.

## 2 PROBLEM FORMULATION

### 2.1 The forward problem of electrocardiography

**FIGURE 1:**
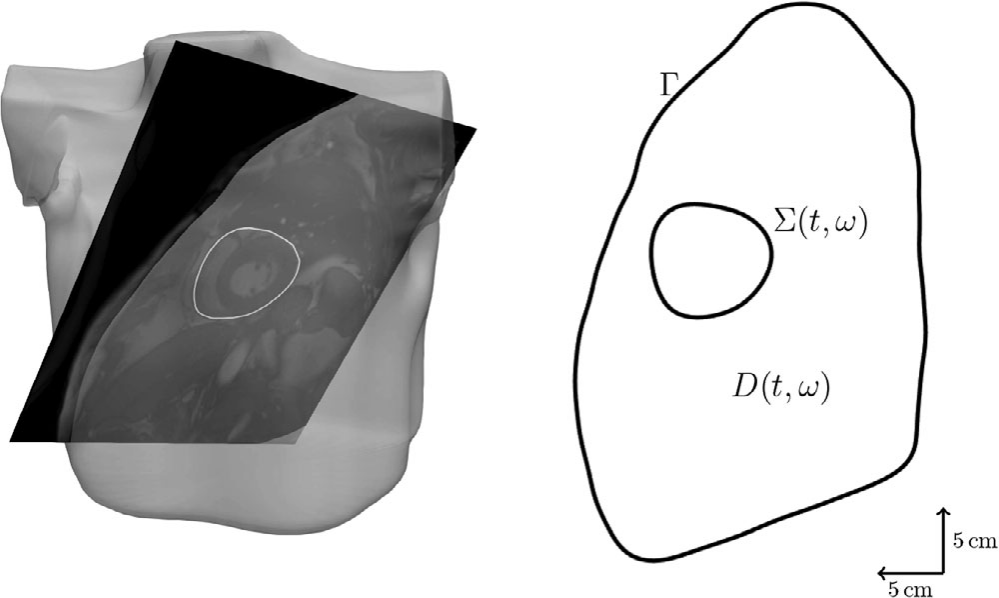
2D cross section of the torso reconstructed from an MRI dataset

Mathematically, we start from a smooth domain $D\subset {\mathbb{R}}^{d}$ representing the torso, whose boundaries are the chest denoted by Γ and the pericardium denoted by ∑. In other words, we have ∂D=∑∪Γ and ∑∩Γ=∅, see Figure 1 for a visualisation of the domain.

The pericardium depends periodically on time and is subject to uncertainty. To model the time‐dependence, let $\left[0,T\right]$ be the time interval of interest, where T>0 is the duration of one heartbeat. To model the uncertainty, let $\left(\Omega ,ℱ,\mathbb{P}\right)$ denote a complete and separable probability space, where Ω is a sample space, $ℱ\subseteq 2^{\Omega }$ is a σ‐field and $\mathbb{P}:ℱ\to \left[0,1\right]$ is a probability measure. In what follows, we write $\sum \left(t,\omega \right)$ with $t\in \left[0,T\right)$ and ω∈Ω to indicate the dependence of the pericardium on time and on the random parameter. Obviously, the time‐dependence and the shape uncertainty of the heart also affect the torso, which we denote by $D\left(t,\omega \right)$, while we assume the chest Γ to be fixed over time and not being subject to uncertainty.

In the forward problem, given the potential $u\left(t\right):\sum \left(t,\omega \right)\to \mathbb{R}$ at the pericardium, we wish to compute the potential on the chest Γ. Assuming that the torso is a homogeneous volume conductor and that no current sources are present, the extracellular potential $y\left(t,\omega \right):D\left(t,\omega \right)\to R$ in the whole torso satisfies the mixed boundary value problem

(1) $$\left\{\begin{array}{llll} \;\Delta y\left(x,t,\omega \right)=0, & \; & x\in D\left(t,\omega \right), & \\ \frac{\partial y}{\partial n_{x}}\left(x,t,\omega \right)=0, & \; & x\in \Gamma , & \\ \;y\left(x,t,\omega \right)=u\left(x,t\right), & \; & x\in \sum \left(t,\omega \right). & \end{array}\right.$$

We remark that in the model the electric conductivity is unit valued, with no loss of generality since its value, being constant, does not influence the solution. Moreover, we assume here that the potential $u\left(x,t\right)$ is given in spatial coordinates. A possibility to guarantee that $u\left(x,t\right)$ is well‐defined for each realisation of the random parameter is to assume that it is given with respect to the hold‐all domain

$$D≔\underset{t\in \left[0,T\right],\omega \in \Omega }{\cup }\bar{D\left(t,\omega \right)}\times \left\{t\right\},$$

which contains every possible space–time tube.

### 2.2 The inverse problem of electrocardiography

Given the data $y_{d}\left(t\right):\Gamma \to \mathbb{R}$ on the chest, the inverse problem corresponding to (1) is to find the potential $u\left(t,\omega \right):\sum \left(t,\omega \right)\to \mathbb{R}$ at the pericardium that satisfies

$$\min_{u\left(t,\omega \right)\in L^{2}\left(\sum \left(t,\omega \right)\right)}∥y\left(t,\omega \right)-y_{d}\left(t\right){∥}_{L^{2}\left(\Gamma \right)}\;s.t.\;\text{Equation}\;\left(1\right)\;\text{holds}.$$

In the forward problem, if $u\left(\cdot ,t\right)\in H^{1/2}\left(\sum \left(t,\omega \right)\right)$, then the solution satisfies $y\left(\cdot ,t,\omega \right)\in H^{1}\left(D\left(t,\omega \right)\right)$ and its trace on Γ is in $H^{1/2}\left(\Gamma \right)$. This gives rise to the solution operator

$$A\left(t,\omega \right):H^{1/2}\left(\sum \left(t,\omega \right)\right)\to H^{1/2}\left(\Gamma \right).$$

It is possible to show, see Reference 3, that $A\left(t,\omega \right)$ is injective and continuous for $t\in \left[0,T\right]$ and almost every ω∈Ω. In the inverse problem, therefore, if $y_{d}\left(\cdot ,t\right)\in H^{1/2}\left(\Gamma \right)$ then there exists a unique minimum $u\left(\cdot ,t,\omega \right)\in H^{1/2}\left(\sum \left(t,\omega \right)\right)$ such that $y\left(t,\omega \right)=y_{d}\left(t\right)$ at Γ. The minimum $u\left(t,\omega \right)$, however, is not stable with respect to perturbations. A common practice is to introduce a regularisation into the problem to recover stability, as follows:

$$\min_{u\left(t,\omega \right)\in L^{2}\left(\sum \left(t,\omega \right)\right)}\left\{\frac{1}{2}∥A\left(t,\omega \right)u\left(t,\omega \right)-y_{d}\left(t\right){∥}_{L^{2}\left(\Gamma \right)}^{2}+\frac{\lambda }{2}ℛ(u(t,\omega ))\right\}.$$

Herein, λ>0 is the regularisation parameter and ℛ is the regularisation functional, for which several choices are possible. The optimum can be explicitly computed according to

$${\langle A\left(t,\omega \right)u\left(t,\omega \right)-y_{d}\left(t\right),A\left(t,\omega \right)\mathrm{\delta u}\rangle }_{L^{2}\left(\Gamma \right)}+\lambda \langle {ℛ}^{\prime }\left(u\left(t,\omega \right)\right),\mathrm{\delta u}\rangle =0,\;\forall \mathrm{\delta u}\in L^{2}\left(\sum \left(t,\omega \right)\right).$$

Later on we shall discuss different options for the regularisation.

## 3 BOUNDARY INTEGRAL FORMULATION

### 3.1 Boundary integral operators

In the following exposition, for the sake of an easier notation, we consider t and ω to be fixed. For a comprehensive exposition of the boundary integral approach, we refer the reader of Reference 12. For a better distinction of quantities that are defined with respect to the boundaries and those which are given on the domain, we introduce for $\Phi \in \left\{\sum ,\Gamma ,\partial D\right\}$ the trace operators

$$\gamma_{0,\Phi }^{\mathrm{int}}:H^{1}\left(D\right)\to H^{1/2}\left(\Phi \right)\;\text{and}\;\gamma_{1,\Phi }^{\mathrm{int}}:H^{1}\left(D\right)\to H^{-1/2}\left(\Phi \right).$$

Then, we may write the forward problem according to

$$\left\{\begin{array}{cc} \Delta y\left(x\right)=0,\; & x\in D,\\ \gamma_{1,\Gamma }^{\mathrm{int}}y\left(x\right)=0,\; & x\in \Gamma ,\\ \gamma_{0,\sum }^{\mathrm{int}}y\left(x\right)=u\left(x\right), & x\in \sum . \end{array}\right.$$

For x∈D, the potential $y\left(x\right)$ is given by the representation formula

(2) $$y\left(x\right)=\int_{\partial D}G\left(x,x^{\prime }\right)\left(\gamma_{1,\partial D}^{\mathrm{int}}y\right)\left(x^{\prime }\right)d\;\sigma_{x^{\prime }}-\int_{\partial D}\frac{\partial G}{\partial n_{x^{\prime }}}\left(x,x^{\prime }\right)\left(\gamma_{0,\partial D}^{\mathrm{int}}y\right)\left(x^{\prime }\right)d\;\sigma_{x^{\prime }},$$

where

(3) $$G\left(x,x^{\prime }\right)≔\left\{\begin{array}{cc} -\frac{1}{2\pi }\log∥x-x^{\prime }{∥}_{2}, & d=2,\\ \;\frac{1}{4\pi }\frac{1}{∥x-x^{\prime }{∥}_{2}}, & d=3, \end{array}\right.$$

denotes the fundamental solution for the Laplacian. Introducing the single layer operator

$$V:H^{-1/2}\left(\partial D\right)\to H^{1/2}\left(\partial D\right),\;\left(V\rho \right)\left(x\right)≔\int_{\partial D}G\left(x,x^{\prime }\right)\rho \left(x^{\prime }\right)d\;\sigma_{x^{\prime }},$$

the double layer operator

$$K:H^{1/2}\left(\partial D\right)\to H^{1/2}\left(\partial D\right),\;\left(K\rho \right)\left(x\right)≔\int_{\partial D}\frac{\partial G}{\partial n_{x^{\prime }}}\left(x,x^{\prime }\right)\rho \left(x^{\prime }\right)d\;\sigma_{x^{\prime }}$$

and taking the Dirichlet trace, Equation (2) yields the Dirichlet‐to‐Neumann map

(4) $$V\left(\gamma_{1,\partial D}^{\mathrm{int}}y\right)=\left(\frac{1}{2}I+K\right)\left(\gamma_{0,\partial D}^{\mathrm{int}}y\right).$$

Next, introducing for $\Phi ,\Psi \in \left\{\sum ,\Gamma \right\}$, the restricted operators

$$V_{\Phi \Psi }:H^{-1/2}\left(\Psi \right)\to H^{1/2}\left(\Phi \right),\;\Phi ∍x\mapsto \left(V_{\Phi \Psi }\rho \right)\left(x\right)≔\int_{\Psi }G\left(x,x^{\prime }\right)\rho \left(x^{\prime }\right)d\;\sigma_{x^{\prime }}$$

and

$$K_{\Phi \Psi }:H^{1/2}\left(\Psi \right)\to H^{1/2}\left(\Phi \right),\;\Phi ∍x\mapsto \left(\Phi_{\Psi }\rho \right)\left(x\right)≔\int_{\Psi }\frac{\partial G}{\partial n_{x'}}\left(x,x^{\prime }\right)\rho \left(x^{\prime }\right)d\;\sigma_{x^{\prime }},$$

we can split up Equation (4) into the system

$$\left[\begin{array}{cc} V_{\sum \sum } & V_{\sum \Gamma }\\ V_{\Gamma \sum } & V_{\Gamma \Gamma } \end{array}\right]\left[\begin{array}{c} \gamma_{1,\sum }^{\mathrm{int}}y\\ \gamma_{1,\Gamma }^{\mathrm{int}}y \end{array}\right]=\left[\begin{array}{cc} \frac{1}{2}I+K_{\sum \sum } & K_{\sum \Gamma }\\ K_{\Gamma \sum } & \frac{1}{2}I+K_{\Gamma \Gamma } \end{array}\right]\left[\begin{array}{c} \gamma_{0,\sum }^{\mathrm{int}}y\\ \gamma_{0,\Gamma }^{\mathrm{int}}y \end{array}\right].$$

Rearranging this system in order to move the unknowns $\gamma_{1,\sum }^{\mathrm{int}}y$ and $\gamma_{0,\Gamma }^{\mathrm{int}}y$ to the left side and the data $\gamma_{0,\sum }^{\mathrm{int}}y=u$ and $\gamma_{1,\Gamma }^{\mathrm{int}}y=0$ to the right side, we arrive at the system boundary integral equations

(5) $$\left[\begin{array}{cc} V_{\sum \sum } & -K_{\sum \Gamma }\\ -V_{\Gamma \sum } & \frac{1}{2}I+K_{\Gamma \Gamma } \end{array}\right]\left[\begin{array}{c} \gamma_{1,\sum }^{\mathrm{int}}y\\ \gamma_{0,\Gamma }^{\mathrm{int}}y \end{array}\right]=\left[\begin{array}{cc} \frac{1}{2}I+K_{\sum \sum } & -V_{\sum \Gamma }\\ -K_{\Gamma \sum } & V_{\Gamma \Gamma } \end{array}\right]\left[\begin{array}{c} u\\ 0 \end{array}\right].$$

As $V_{\sum \sum }$ is an elliptic operator, considering the Schur complement

$$S:H^{1/2}\left(\Gamma \right)\to H^{1/2}\left(\Gamma \right),\;S\rho ≔\left(\frac{1}{2}I+K_{\Gamma \Gamma }-V_{\Gamma \sum }V_{\sum \sum }^{-1}K_{\sum \Gamma }\right)\rho$$

yields the operator equation

$$S\gamma_{0,\Gamma }^{\mathrm{int}}y=\left(\frac{1}{2}V_{\Gamma \sum }V_{\sum \sum }^{-1}+V_{\Gamma \sum }V_{\sum \sum }^{-1}K_{\sum \sum }-K_{\Gamma \sum }\right)u$$

for the desired potential $\gamma_{0,\Gamma }^{\mathrm{int}}y$ on the chest. In particular, the solution operator $A:H^{1/2}\left(\sum \right)\to H^{1/2}\left(\Gamma \right)$ is given by

(6) $$Au=S^{-1}\left(\frac{1}{2}V_{\Gamma \sum }V_{\sum \sum }^{-1}+V_{\Gamma \sum }V_{\sum \sum }^{-1}K_{\sum \sum }-K_{\Gamma \sum }\right)u=\gamma_{0,\Gamma }^{\mathrm{int}}y,$$

while the Poincaré–Steklov operator $ℬ:H^{1/2}\left(\sum \right)\to H^{-1/2}\left(\sum \right)$ is given by

(7) $$ℬu=V_{\sum \sum }^{-1}\left(\frac{1}{2}I+K_{\sum \sum }+K_{\sum \Gamma }A\right)u=\gamma_{1,\sum }^{\mathrm{int}}y.$$

### 3.2 Numerical discretisation

Our goal is to discretise the boundary integral equations derived previously. In order to not having to deal with non‐constant coefficients, we shall solve the equations in the spatial coordinate frame, that is, for each tuple $\left(t,\omega \right)$, assemble the boundary integral operators on $\sum \left(t,\omega \right)$ and Γ. To simplify the notation, in the following quantities we omit the dependency of ∑ on t and ω. As the focus of this work is on uncertainty quantification, rather than on the solution of the spatial problem, we restrict ourselves to the simplified 2D anatomy of Figure 1. Hence, from (3), we have

$$G\left(x,x^{\prime }\right)=-\frac{1}{2\pi }\log∥x-x^{\prime }{∥}_{2}$$

and

$$\frac{\partial G}{\partial n_{x^{'}}}\left(x,x^{\prime }\right)=-\frac{1}{2\pi }\nabla_{{}^{x'}}\log∥x-x^{\prime }{∥}_{2}\cdot n_{x^{'}}=\frac{1}{2\pi }\frac{{\langle x-x^{\prime },n_{x^{'}}\rangle }_{2}}{∥x-x^{\prime }{∥}_{2}^{2}}.$$

Given that the domain $D\left(t,\omega \right)$ exhibits $C^{2}$‐boundaries, which are described by the parameterisations $\gamma_{\sum }:\left[0,1\right)\to \sum \left(t,\omega \right)$ and $\gamma_{\Gamma }:\left[0,1\right)\to \Gamma$, we can employ the collocation method from Reference 12. This method is based on the trapezoidal rule and an appropriate desingularisation of the kernel functions. In view of the Euler‐Maclaurin formula, it converges quadratically for $C^{2}$‐boundaries and exponentially for smooth boundaries. More precisely, given that the solution to (5) satisfies $y\in C^{k}\left(\partial D\right)$, there holds

$$∥y-y_{n}{∥}_{L^{\infty }\left(\partial D\right)}\leq {\mathrm{Cn}}^{-k}∥y{∥}_{C^{k}\left(\partial D\right)}$$

for some C>0, where $y_{n}$ is obtained from the collocation method with n=2j points for some j∈ℕ. We refer to Reference 12 for all the details. For the sake of completeness, we briefly recall the collocation method in Appendix A.

For $\Phi \in \left\{\sum ,\Gamma \right\}$, we consider the $n_{\Phi }$‐points discretisations $s_{i,\Phi }=i/n_{\Phi }$ for $i=0,\ldots ,n_{\Phi }-1$. Moreover, we denote by $I_{n_{\Phi }}\in {\mathbb{R}}^{n_{\Phi }\times n_{\Phi }}$ the $n_{\Phi }\times n_{\Phi }$ identity matrix. The discretisation results in the block linear system

(8) $$\left[\begin{array}{cc} V_{\sum \sum } & -K_{\sum \Gamma }\\ -V_{\Gamma \sum } & \frac{1}{2}I_{n_{\Gamma }}+K_{\Gamma \Gamma } \end{array}\right]\left[\begin{array}{c} {\tilde{\rho }}_{1,\sum }\\ \rho_{0,\Gamma } \end{array}\right]=\left[\begin{array}{cc} \frac{1}{2}I_{n_{\sum }}+K_{\sum \sum } & -V_{\sum \Gamma }\\ -K_{\Gamma \sum } & V_{\Gamma \Gamma } \end{array}\right]\left[\begin{array}{c} u\\ 0_{n_{\Gamma }} \end{array}\right],$$

cp. (5). Herein

$$\rho_{0,\Gamma }≔{\left[y\left(\gamma_{\Gamma }\left(s_{i,\Gamma }\right)\right)\right]}_{i=0}^{n_{\Gamma }-1}\in {\mathbb{R}}^{n_{\Gamma }}\;\text{and}\;{\tilde{\rho }}_{1,\sum }≔{\left[\frac{\partial y}{\partial n_{s_{i},\sum }}\left(\gamma_{\sum }\left(s_{i,\sum }\right)\right)∥\gamma_{\sum }^{\prime }\left(s_{i,\sum }\right){∥}_{2}\right]}_{i=0}^{n_{\sum }-1}\in {\mathbb{R}}^{n_{\sum }}.$$

Furthermore, we set

$$u≔{\left[u\left(\gamma_{\sum }\left(s_{i,\sum }\right)\right)\right]}_{i=0}^{n_{\sum }-1}\in {\mathbb{R}}^{n_{\sum }}$$

and $0_{n_{\Gamma }}\in {\mathbb{R}}^{n_{\Gamma }}$ is the $n_{\Gamma }$‐dimensional vector of zeros.

## 4 SOLUTION OF THE INVERSE PROBLEM

In the inverse problem, the goal is to reconstruct the potential u at the pericardium $\sum \left(t,\omega \right)$ given noisy data measurements $y_{d}\in H^{1/2}\left(\Gamma \right)$ on the chest. In the following exposition, for the sake of an easier notation, we indicate the dependency of ∑ on the tuple $\left(t,\omega \right)$ only when specifying function spaces. In particular, we consider the solution operator $A:H^{1/2}\left(\sum \left(t,\omega \right)\right)\to H^{1/2}\left(\Gamma \right)$, cp. (6), and the Poincaré–Steklov operator $ℬ:H^{1/2}\left(\sum \left(t,\omega \right)\right)\to H^{-1/2}\left(\sum \left(t,\omega \right)\right)$, cp. (7). In the discrete setting, we define the vectors

$$u≔{\left[u\left(\gamma_{\sum }\left(s_{i,\sum }\right)\right)\right]}_{i=0}^{n_{\sum }-1}\in {\mathbb{R}}^{n_{\sum }},\;y_{d}≔{\left[y_{d}\left(\gamma_{\Gamma }\left(s_{i,\Gamma }\right)\right)\right]}_{i=0}^{n_{\Gamma }-1}\in {\mathbb{R}}^{n_{\Gamma }}$$

and

$$\rho_{0,\Gamma }≔{\left[y\left(\gamma_{\Gamma }\left(s_{i,\Gamma }\right)\right)\right]}_{i=0}^{n_{\Gamma }-1}\in {\mathbb{R}}^{n_{\Gamma }},\;\rho_{1,\sum }≔{\left[\frac{\partial y}{\partial n_{s_{i},\sum }}\left(\gamma_{\sum }\left(s_{i,\sum }\right)\right)\right]}_{i=0}^{n_{\sum }-1}\in {\mathbb{R}}^{n_{\sum }}.$$

From (8), we deduce that there exist a matrix $A\in {\mathbb{R}}^{n_{\Gamma }\times n_{\sum }}$ and a matrix $B\in {\mathbb{R}}^{n_{\sum }\times n_{\sum }}$ such that

$$\rho_{0,\Gamma }=\mathrm{Au}\;\text{and}\;\rho_{1,\sum }=\mathrm{Bu}.$$

In this section, we consider inverse problem solutions of the form

(9) $$u=\underset{v\in L^{2}\left(\sum \left(t,\omega \right)\right)}{\text{argmin}}\left\{\frac{1}{2}∥Av-y_{d}{∥}_{L^{2}\left(\Gamma \right)}^{2}+\frac{\lambda }{2}ℛ\left(v\right)\right\},$$

where λ>0 is the regularisation parameter and ℛ is the regularisation functional. We discretise (9) employing, for $\Phi \in \left\{\sum ,\Gamma \right\}$, the trapezoidal rule

$${\langle v,w\rangle }_{L^{2}\left(\Phi \right)}\approx v^{⊺}S_{\Phi }w\;\text{with}\;v≔{\left[v\left(\gamma_{\Phi }\left(s_{i,\Phi }\right)\right)\right]}_{i=0}^{n_{\Phi }-1},\;w≔{\left[w\left(\gamma_{\Phi }\left(s_{i,\Phi }\right)\right)\right]}_{i=0}^{n_{\Phi }-1},$$

where $S_{\Phi }\in {\mathbb{R}}^{n_{\Phi }\times n_{\Phi }}$ is the diagonal mass matrix with entries

$${\left(S_{\Phi }\right)}_{i,i}≔\frac{∥\gamma_{\Phi }^{'}\left(s_{i,\Phi }\right){∥}_{2}}{n_{\Phi }}$$

for $i=0,\ldots ,n_{\Phi }-1$. The accuracy of this approximation is consistent with the one obtained by the collocation method, as can easily be inferred from the Euler‐Maclaurin formula.

We arrive at the discrete formulation

(10) $$u=\underset{v\in {\mathbb{R}}^{n_{\sum }}}{\text{argmin}}\left\{\frac{1}{2}{\left(\mathrm{Av}-y_{d}\right)}^{⊺}S_{\Gamma }\left(\mathrm{Av}-y_{d}\right)+\frac{\lambda }{2}R\left(v\right)\right\},$$

where R is the discrete regularisation term. The optimisation of the objective function leads to the equation

(11) $$A^{⊺}S_{\Gamma }\left(\mathrm{Au}-y_{d}\right)+\frac{\lambda }{2}R^{\prime }\left(u\right)=0$$

for the minimum u. In the following subsections, we present four strategies to solve the inverse problem based on different regularisations.

### 4.1 Zero order Tikhonov regularisation

The zero order Tikhonov regularisation penalises the $L^{2}$‐norm of the Dirichlet data at $\sum \left(t,\omega \right)$, see Reference 34, and the regularisation functional in (9) reads

$$ℛ\left(v\right)=∥v{∥}_{L^{2}\left(\sum \left(t,\omega \right)\right)}^{2}.$$

The discrete regularisation term in (10) is thus

$$R\left(v\right)=v^{⊺}S_{\sum }v$$

and in (11) we have

$$R^{\prime }\left(u\right)=2S_{\sum }u.$$

### 4.2 First‐order Tikhonov regularisation

The first‐order Tikhonov regularisation penalises the $L^{2}$‐norm of the Neumann data at $\sum \left(t,\omega \right)$, see Reference 34, and the regularisation functional in (9) is

$$ℛ\left(v\right)=∥ℬv{∥}_{L^{2}\left(\sum \left(t,\omega \right)\right)}^{2}.$$

Then the discrete regularisation term in (10) is

$$R\left(v\right)=v^{⊺}B^{⊺}S_{\sum }\mathrm{Bv}$$

and in (11) we have

$$R^{\prime }\left(u\right)=2B^{⊺}S_{\sum }\mathrm{Bu}.$$

### 4.3 $H^{1/2}$ regularisation

When penalising the $H^{1/2}$‐norm of the Dirichlet data at $\sum \left(t,\omega \right)$, the regularisation functional in (9) is given by

$$ℛ\left(v\right)=∥v{∥}_{H^{1/2}\left(\sum \left(t,\omega \right)\right)}^{2}={\langle ℬv,v\rangle }_{L^{2}\left(\sum \left(t,\omega \right)\right)}.$$

The resulting discrete regularisation term in (10) is

$$R\left(v\right)=v^{⊺}B^{⊺}S_{\sum }v$$

and its Fréchet derivative in (11) is

$$R^{'}\left(u\right)=S_{\sum }\left(B^{⊺}+B\right)u.$$

### 4.4 Total variation regularisation

In the TV regularisation, we penalise the $L^{1}$‐norm of the Neumann data at $\sum \left(t,\omega \right)$, that is, the regularisation functional in (9) is

$$ℛ\left(v\right)=∥ℬv{∥}_{L^{1}\left(\sum \left(t,\omega \right)\right)}.$$

As the $L^{1}$‐norm is non‐differentiable, it is common to employ the approximation

$$∥ℬv{∥}_{L^{1}\left(\sum \left(t,\omega \right)\right)}=\int_{\sum \left(t,\omega \right)}|ℬv\left(x\right)|d\sigma_{x}\approx \int_{\sum \left(t,\omega \right)}\sqrt{{\left(ℬv\left(x\right)\right)}^{2}+\beta }\;d\sigma_{x},$$

where β>0 is a small constant (typically $\beta =10^{-5}$). This introduces a non‐linearity in the optimality condition. This difficulty is handled in Reference 4 by using the zero order Tikhonov solution $u_{0}$ in the non‐linear term. This approach is equivalent to set

$$ℛ\left(v\right)=\int_{\sum \left(t,\omega \right)}{\left(2\sqrt{{\left(ℬu_{0}\left(x\right)\right)}^{2}+\beta }\right)}^{-1}{\left(ℬv\left(x\right)\right)}^{2}d\;\sigma_{x}$$

in (9). The discretisation requires the definition of the diagonal matrix $W_{\beta }\left(u_{0}\right)\in {\mathbb{R}}^{n_{\sum }\times n_{\sum }}$ with entries

$${\left(W_{\beta }\left(u_{0}\right)\right)}_{i,i}≔{\left(2\sqrt{{\left({\mathrm{Bu}}_{0}\right)}_{i}^{2}+\beta }\right)}^{-1}$$

for $i=0,\ldots ,n_{\sum }-1$, where $u_{0}$ is the discrete zero order Tikhonov solution. The discrete regularisation term in (10) then reads

$$R\left(v\right)=v^{⊺}B^{⊺}W_{\beta }\left(u_{0}\right)S_{\sum }\mathrm{Bv},$$

while in (11) it results in

$$R^{'}\left(u\right)=2B^{⊺}W_{\beta }\left(u_{0}\right)S_{\sum }\mathrm{Bu}.$$

## 5 UNCERTAINTY QUANTIFICATION

### 5.1 Modelling time‐dependent shape uncertainty

In order to assess the shape uncertainty of the pericardium $\sum \left(t,\omega \right)$, we assume the existence of a reference domain $D_{\mathrm{ref}}\subset {\mathbb{R}}^{d}$, with boundaries $\sum_{\mathrm{ref}}$ and Γ, and a random deformation field

$$\chi :D_{\mathrm{ref}}\times \left[0,T\right]\times \Omega \to {\mathbb{R}}^{d}$$

such that

$$D\left(t,\omega \right)=\chi \left(D_{\mathrm{ref}},t,\omega \right)=\chi \left(D_{\mathrm{ref}}\left(t\right),\omega \right),$$

where

$$D_{\mathrm{ref}}\left(t\right)=E\left[\chi \right]\left(D_{\mathrm{ref}},t\right).$$

Herein, the expectation is given in terms of the Bochner integral

$$E\left[\chi \right]\left(\hat{x},t\right)≔\int_{\Omega }\chi \left(\hat{x},t,\omega \right)\mathrm{d\mathbb{P}}.$$

Introducing the space–time cylinder $Q_{\mathrm{ref}}≔D_{\mathrm{ref}}\times \left(0,T\right)$ with coordinates

$$\left(\hat{x},t\right)≕\;\hat{z}\in Q_{\mathrm{ref}},$$

we can interpret χ as a random deformation field $\chi \left(\hat{z},\omega \right)$ acting on the space–time cylinder. A similar model has originally been introduced in Reference 25 for the stationary case. Assuming

$$\chi \in L^{2}\left(\Omega ;C^{1}\left(Q_{\mathrm{ref}};{\mathbb{R}}^{d}\right)\right),$$

it is possible to expand $\chi \left(\hat{z},\omega \right)$ in a Karhunen–Loève expansion, cf., Reference 35 according to

$$\chi \left(\hat{z},\omega \right)=E\left[\chi \right]\left(\hat{z}\right)+\sum_{k=1}^{\infty }\sqrt{\lambda_{k}}\chi_{k}\left(\hat{z}\right)Y_{k}\left(\omega \right).$$

Herein, $\left(\lambda_{k},\chi_{k}\right)$ are the eigen‐pairs of the integral operator defined by the matrix valued covariance function

$$\mathrm{Cov}\left[\chi \right]:Q_{\mathrm{ref}}\times Q_{\mathrm{ref}}\to {\mathbb{R}}^{d\times d},\mathrm{Cov}\left[\chi \right]\left(\hat{z},\hat{z^{\prime }}\right)≔\int_{\Omega }\left(\chi \left(\hat{z},\omega \right)-E\left[\chi \right]\left(\hat{z}\right)\right){\left(\chi \left(\hat{z^{\prime }},\omega \right)-E\left[\chi \right]\left(\hat{z^{\prime }}\right)\right)}^{⊺}\mathrm{d\mathbb{P}},$$

while $\left\{Y_{k}\right\}$ is a family of uncorrelated random variables with normalised variance.

To avoid distortion of the space–time tubes $\chi \left(Q_{\mathrm{ref}},\omega \right)$, ω∈Ω, and hence to guarantee well‐posedness of the boundary value problem at hand, cf. (1), we impose the uniformity condition

$$∥\chi \left(\cdot ,\omega \right){∥}_{C^{1}\left(\bar{Q_{\mathrm{ref}}};{\mathbb{R}}^{d}\right)},∥\chi^{-1}\left(\cdot ,\omega \right){∥}_{C^{1}\left(\bar{Q\left(\omega \right)};{\mathbb{R}}^{d}\right)}<C_{\mathrm{uni}}$$

for some constant $C_{\mathrm{uni}}>0$ uniformly in ω∈Ω.^1^ As a consequence, the random variables' $\left\{Y_{k}\right\}$ ranges are bounded. Therefore, without loss of generality we may assume $Y_{k}:\Omega \to \left[-1,1\right]$, k=1,2,…. In particular, we obtain

$$\sum \left(t,\omega \right)=\chi \left(\sum_{\mathrm{ref}},t,\omega \right)\;\text{and}\;\Gamma =\chi \left(\Gamma ,t,\omega \right).$$

Replacing the random variables by their image ${\left[-1,1\right]}^{\mathbb{N}}$, we arrive at the parametrised Karhunen–Loève expansion

$$\chi \left(\hat{z},\xi \right)=E\left[\chi \right]\left(\hat{z}\right)+\sum_{k=1}^{\infty }\sqrt{\lambda_{k}}\chi_{k}\left(\hat{z}\right)\xi_{k},\;\xi \in {\left[-1,1\right]}^{\mathbb{N}}.$$

For the numerical computation of the Karhunen–Loève expansion, it is sufficient to know the expectation and the covariance at $\sum_{\mathrm{ref}}\times \left[0,T\right]$, see Reference 36. In our concrete case, it is even sufficient to only compute the Karhunen–Loève expansion in the space–time collocation points. To this end, we employ the pivoted Cholesky decomposition, see References 25, 37, 38. These results in a finite rank Karhunen–Loève expansion

(12) $$\chi \left({\hat{z}}_{i},\xi \right)=E\left[\chi \right]\left({\hat{z}}_{i}\right)+\sum_{k=1}^{K}\sqrt{\lambda_{k}}\chi_{k}\left({\hat{z}}_{i}\right)\xi_{k},\;\xi \in {\left[-1,1\right]}^{K},$$

where ${\hat{z}}_{i}$ are the space–time collocation points on $\sum_{\mathrm{ref}}\times \left[0,T\right]$ and K is the dimension of the random parameter space.

### 5.2 Computation of quantities of interest

We make the common assumption that the random variables are even independent and uniformly distributed. Then, the push‐forward measure is given by the product density

$$\rho \left(\xi \right)≔\prod_{k=1}^{K}\rho_{k}\left(\xi_{k}\right)\;\text{with}\;\rho_{k}\equiv \frac{1}{2}.$$

Hence, we can now express quantities of interest, such as the moments of the potential on the chest, by means of an integral with respect to ${\left[-1,1\right]}^{K}$. It holds for the moments that

$${ℳ}_{m}\left[y\right]\left(x,t\right)≔\int_{\Omega }\;y{\left(x,t,\omega \right)}^{m}d\;\mathbb{P}=\int_{{\left[-1,1\right]}^{K}}y{\left(x,t,\xi \right)}^{m}\rho \left(\xi \right)d\;\xi ,\;m\in {\mathbb{N}}^{*}.$$

Especially, the expectation and the variance are given by

$$E\left[y\right]\left(x,t\right)={ℳ}_{1}\left[y\right]\left(x,t\right)\;\text{and}\;V\left[y\right]\left(x,t\right)={ℳ}_{2}\left[y\right]\left(x,t\right)-{\left({ℳ}_{1}\left[y\right]\left(x,t\right)\right)}^{2}.$$

In practice, the moments need to be approximated by a quadrature rule, that is,

$${ℳ}_{m}\left[y\right]\left(x,t\right)\approx \sum_{i=1}^{N}w_{i}y{\left(x,t,\xi_{i}\right)}^{m},$$

where $\xi_{i}\in {\left[-1,1\right]}^{K}$ for i=1,…,N are the quadrature points and $w_{i}\in \mathbb{R}$ are the corresponding weights. Due to the space–time modelling, the random parameter space is very high‐dimensional, and appropriate quadrature rules need to be employed. In this article, we employ the anisotropic sparse quadrature from Reference 28, which is based on the Gauss–Legendre quadrature rule. For the sparse quadrature to converge, the quantity of interest needs to be regular with respect to the random parameter $\xi \in {\left[-1,1\right]}^{K}$. For the forward problem, such results are available, we refer to Reference 25. For the Dirichlet control problem with an affine random diffusion coefficient, such results have also been derived in Reference 39.

## 6 NUMERICAL EXPERIMENTS

In this section, we present numerical experiments to assess the validity of the approach. To obtain a space–time reference torso anatomy, we have segmented cardiac magnetic resonance (CMR) imaging data previously acquired.^40^ The temporal image stack was obtained from a cine ECG‐triggered segmented steady‐state free precession sequence in mid‐ventricular short‐axis orientation. Slice thickness and voxel resolution were 8 and 1.36 mm, respectively. The 25‐phase temporal stack covered the whole heartbeat, with an RR interval on the surface ECG of T=690 ms. Images were reordered such that the first image at t=0 ms corresponded to the diastole, defined by the maximal left ventricular cavity volume. The systole, defined by the minimum cavity volume, occurred at t=270 ms. The segmentation was performed by manual contour tracing of the epicardium for each image. In order to end up with a smooth computational domain, we performed a least‐squares fit of the contours using a truncated Fourier series with a threshold of $10^{-3}$ in the relative root‐mean‐square error. Finally, we interpolated the extracted shapes to get a pericardial representation at 50 time instants. The chest was also previously segmented from ultra‐fast gradient‐echo “VIBE” images in axial, coronal, and sagittal orientations to produce a smooth three‐dimensional closed surface modelled in Blender.^2^ As shown in Figure 1, the contour of the chest was eventually obtained by intersecting the chest surface with the orientation plane of the pericardium.

**FIGURE 2:**
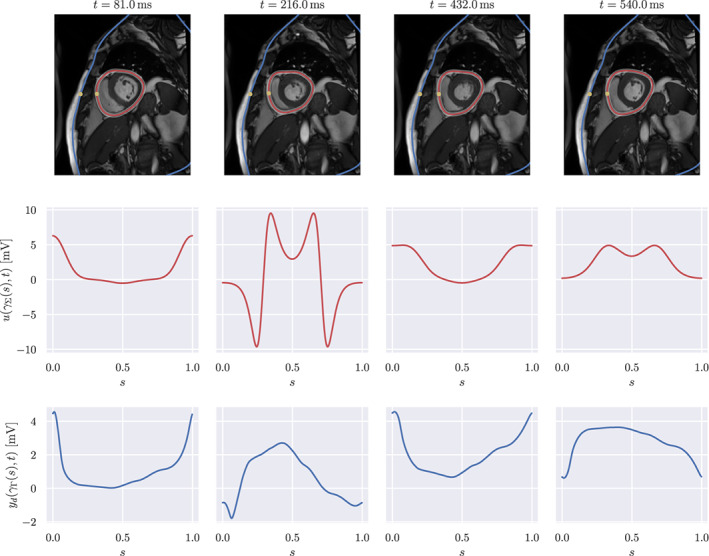
Geometry and input data. First row: cardiac magnetic resonance images with superimposed segmented chest (blue) and time‐dependent pericardium (red). The shaded region around the pericardium represents the shape confidence interval, obtained as $E\left[\sum \right]\left(t\right)\pm 1.96\cdot \text{StdDev}\left[\sum \right]\left(t\right)$. The yellow dots correspond to $\gamma_{\Gamma }\left(0\right)$ and $\gamma_{\sum_{\mathrm{ref}}\left(t\right)}\left(0\right)$. Second row: forward data $u\left(\gamma_{\sum }\left(s\right),t\right)$. Third row: inverse data $y_{d}\left(\gamma_{\Gamma }\left(s\right),t\right)$

In Figure 2, we summarised the input data for the forward and inverse problem and the geometry of the pericardium, superimposed with the CMR images. To model the uncertainty, we selected the resulting reference shape of the pericardium $\sum_{\mathrm{ref}}\left(t\right)$, for $t\in \left[0,T\right)$, as the space–time mean of the random deformation field χ. The covariance of χ evaluated at the points $\hat{z}=\left(\hat{x},t\right)$ with $\hat{x}\in \sum_{\mathrm{ref}}\left(t\right)$ and ${\hat{z}}^{\prime }=\left({\hat{x}}^{\prime },t^{\prime }\right)$ with ${\hat{x}}^{\prime }\in \sum_{\mathrm{ref}}\left(t^{\prime }\right)$ was the product of a matrix‐valued kernel in space and a scalar kernel in time. For the spatial component of the covariance, we considered the Matérn kernel

$$k_{\nu }^{M}\left(d\right)=\sigma^{2}\frac{2^{1-\nu }}{\Gamma \left(\nu \right)}{\left(\sqrt{2\nu }\frac{d}{\rho }\right)}^{\nu }K_{\nu }\left(\sqrt{2\nu }\frac{d}{\rho }\right),$$

with parameters ρ=50, $\sigma^{2}=4/3$ and different values of the smoothness index ν. Note that in the definition of $k_{\nu }^{M}$, the function $\Gamma \left(\cdot \right)$ is the gamma function and $K_{\nu }\left(\cdot \right)$ is the modified Bessel function of the second kind. Since we are interested in modelling the uncertainty due to the noise and the limited resolution of CMR, the distance $d=d\left(\hat{x},\hat{x^{\prime }}\right)$ used in the definition of the kernel, was measured with the Euclidean norm in ${\mathbb{R}}^{2}$. In contrast, for the distance in time between t and $t^{'}$, we take into account the periodicity of the motion of the pericardium. To do so, we first scale the interval $\left[0,T\right)$ to $\left[0,2\pi \right)$ with the mapping

$$\eta :\left[0,T\right)\to \left[0,2\pi \right),\;t\mapsto \frac{2\mathrm{\pi t}}{T},$$

and then we compute the geodesic distance

$$\theta \left(t,t^{'}\right)=\arccos\left(\cos\left(\right.\eta \left(t\right)-\eta \left(\right.t^{'}))\right).$$

Finally, to model an additional correlation between neighbouring time slices, we employ the sine power kernel

$$k^{\mathrm{SP}}\left(\theta \right)=1-{\left(\sin\left(\theta /2\right)\right)}^{2}={\left(\cos\left(\theta /2\right)\right)}^{2}=\frac{1}{2}\left(\cos\left(\theta \right)+1\right),$$

see Reference 41. Employing a tensor product construction, we end up with the covariance function

$$\mathrm{Cov}\left[\chi \right]\left(\hat{z},\hat{z^{\prime }}\right)=k^{\mathrm{SP}}\left(\theta \left(t,t^{\prime }\right)\right)\left[\begin{array}{cc} k_{5/2}^{M}\left(∥\hat{x}\left(t\right)-\hat{x^{\prime }}\left(t^{\prime }\right){∥}_{2}\right) & 0\\ 0 & k_{\infty }^{M}\left(∥\hat{x}\left(t\right)-\hat{x^{\prime }}\left(t^{\prime }\right){∥}_{2}\right) \end{array}\right].$$

Note that we assume here that there is no correlation between the two spatial components of the deformation field. By construction, the joint covariance kernel is positive‐definite on the space ${\mathbb{R}}^{2}\times S^{1}$, where $S^{1}$ is the unit circle.

In the forward problem, the pericardial potential $u\left(x,t\right)$ was defined analytically as a T‐periodic function in the variable t. For convenience, we set $u\left(x,t\right)=u\left(\gamma_{\sum \left(t,\omega \right)}\left(s\right),t\right)$, being $s\in \left[0,1\right)$ the normalised curvilinear coordinate. We simulated a left bundle branch block, that is the extracellular potential consisted in a propagation from free wall of the right ventricle, at s=0, towards the free wall of the left ventricle, at s=0.5. The propagation took 150 ms, consistent with a long QRS complex. The specific analytical form of $u\left(x,t\right)$ is given in Appendix B.

To generate the input data for the inverse problem, we computed the solution of the forward problem on the space–time reference geometry and eventually added Gaussian noise with zero mean and variance $10^{-8}$, corresponding to a signal‐to‐noise ratio of approximately 46 dB.

Concerning the space discretisation, we considered $n_{\sum }=n_{\Gamma }=500$ collocation points. This resulted in a truncated Karhunen–Loève expansion with K=648 terms, obtained with a tolerance of $10^{-4}$. That is, the parametric dimension of the UQ problem was 648, thus high‐dimensional. We remark that, since the boundaries are represented by trigonometric polynomials and all data are smooth functions, the collocation method converges exponentially. Resulting in spatial approximation errors for the chosen number of boundary points, which are already of the order of the machine precision.

**FIGURE 3:**
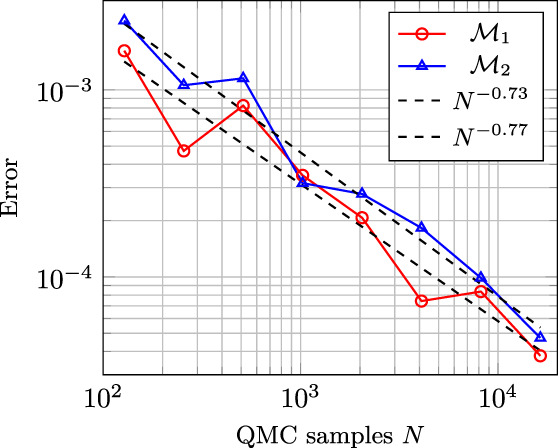
Convergence plot of the first (${ℳ}_{1}$) and second (${ℳ}_{2}$) moment of the forward solution

Since we wish to employ the sparse quadrature to estimate our quantities of interest, we first numerically tested its convergence by comparing it to the quasi‐Monte Carlo method based on the Halton set, see Reference 32. We tested the convergence of the first and second moment of the forward and inverse solution at t=189 ms. The time‐independent problem yielded a truncated Karhunen–Loève expansion of K=101 terms. To validate the applicability of the sparse quadrature, we consider the approximation error of the moments by a comparison to the quasi‐Monte Carlo method based on Halton points. The accuracy of approximately $5\cdot 10^{-5}$ was achieved by using 17'799 quadrature points within the sparse quadrature. Figure 3 shows the convergence plot of the forward solution. We experimentally observed a convergence rate of 0.75. The obtained result indeed corroborates that the forward problem is smooth in the parametric space.

For the inverse problem, we tested the regularisations reported in section 4. The regularisation parameter was determined with the L‐curve method, see Reference 34, on the space–time reference geometry. For each regularisation, we then chose the maximal regularisation parameter over all the time steps. This might have led to over‐regularisation for some time steps, but it has the benefit of limiting the oscillations in the numerical solution of the inverse problem. The values of the chosen regularisation parameters λ are reported in Table 1.

**TABLE 1:** Regularisation parameter choice for the zero‐order Tikhonov, first‐order Tikhonov, $H^{1/2}$ and total variation regularisations

	Zero‐order Tikhonov	First‐order Tikhonov	$H^{1/2}$	Total variation
λ	$10^{-6}$	$10^{-3}$	$10^{-5}$	$10^{-5}$

**FIGURE 4:**
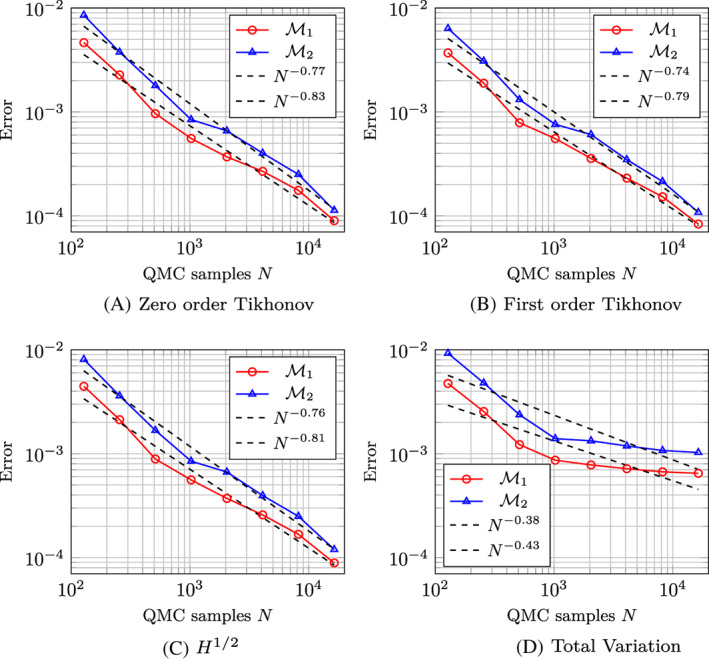
Convergence plot of the first (${ℳ}_{1}$) and second (${ℳ}_{2}$) moment of the inverse solution with (A) zero‐order Tikhonov, (B) first‐order Tikhonov, (C) $H^{1/2}$ and (D) total variation regularisation

Figure 4 shows the convergence plots of the inverse solutions. We can observe convergence towards the reference moments for the zero order Tikhonov, the first‐order Tikhonov and the $H^{1/2}$ regularisations. Instead, the curves resulting from the TV regularisation flatten out, meaning that there is no convergence and suggesting that this regularisation is not sufficiently smooth with respect to the random parameter, and therefore inadequate for the sparse quadrature approach. In contrast, the other regularisations are to be suitable for the sparse quadrature approach.

**FIGURE 5:**
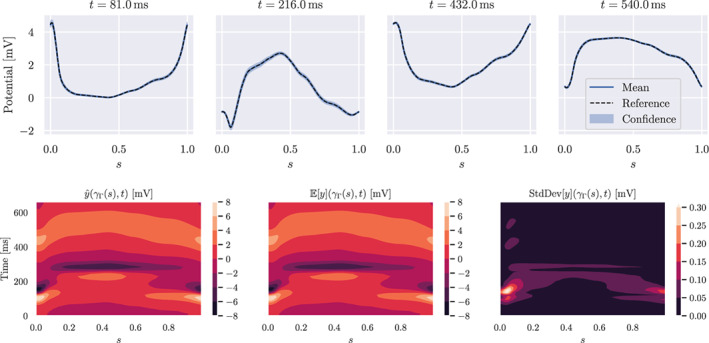
Solution of the forward problem. First row: expectation and confidence interval (blue), and solution with reference geometry (dashed black). Second row: contour plots in space–time of the reference solution, expected solution and standard deviation

Next, we present the corresponding numerical results for the inverse problem, when employing the $H^{1/2}$ regularisation. We estimate the mean and the standard deviation of the forward and inverse solution. The computed quantities of interest for the forward problem are shown in Figure 5. We can observe that the expectation was very close to the chest potential computed on the space–time reference geometry. Moreover, the standard deviation is small and it mainly affects the regions with higher amplitude during the depolarisation phase. In particular, the standard deviation is higher close to $\gamma_{\Gamma }\left(0\right)$. The reason for this phenomenon is that points in the vicinity of $\gamma_{\Gamma }\left(0\right)$ are close to the heart, see Figure 2. Therefore the effect of the shape uncertainty on the forward solution is limited.

**FIGURE 6:**
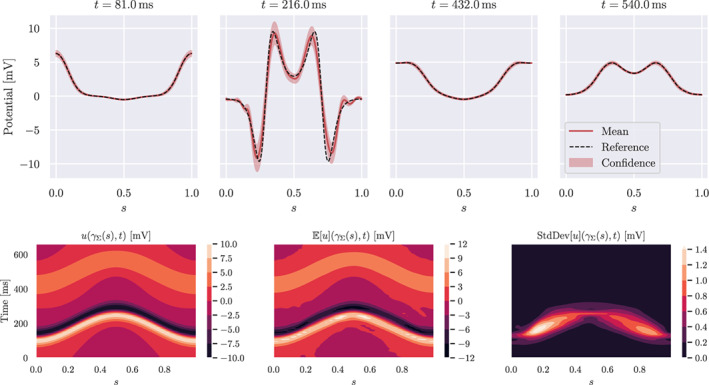
Solution of the inverse problem. First row: expectation and confidence interval (red), and reference solution (dashed black). Second row: contour plots in space–time of the reference solution, expected solution and standard deviation

The computed quantities of interest for the inverse problem are shown in Figure 6. We observe that the expectation deviates slightly from the pericardial potential and shows some oscillations in the depolarisation phase. This is due to the ill‐posedness of the inverse problem and the effect of the regularisation. Indeed, the Tikhonov regularisation, especially at lower order, tends to introduce oscillations in the solution, if the regularisation parameter is not sufficiently large. On the other hand, a strong regularisation yields smoothed out gradients, thus less accurate inverse solution. The $H^{1/2}$ regularisation might therefore introduce oscillations as well. In the bottom row of Figure 6, the standard deviation is larger than in the forward problem and the shape uncertainty mainly affects regions with large gradients. In the depolarisation phase, the potential is steeper and, consequently, the standard deviation is larger. This fact may have a consequence on the quality of derived quantities such as the activation map, usually obtained from the point with largest negative deflection in the signals. In conclusion, the effect of the shape uncertainty is much more relevant for the inverse problem than for the forward problem.

## 7 CONCLUSIONS

In this work, we have considered the forward and inverse problem of electrocardiography in the presence of space–time shape uncertainties. The high parametric dimensionality of the problem, along with the non‐linear map between the input and output uncertainties, renders the problem challenging from the mathematical and the numerical perspective.

To address space–time shape uncertainties, we have suggested a model by random space–time deformation fields with a periodic‐in‐time covariance kernel. This approach resulted in a high‐dimensional uncertainty quantification problem. To make this dimensionality feasible for numerical computations, we rely on the one hand on a low‐rank representation of the random deformation field and efficient quadrature techniques on the other hand. The resulting spatial problem for each quadrature point in the parameter was addressed by a boundary integral formulation in combination with a highly accurate and fast collocation method. The numerical results indicate that shape uncertainties affect the solution much more strongly in the inverse problem than in the forward one, as a consequence of the ill‐posedness. Moreover, it was observed that the regularisation of the inverse solution may affect the regularity of the solution with respect to the stochastic parameter, with possible limitations in the convergence order of the quadrature method. Especially, the TV regularisation showed poor performance in this respect, while the $H^{1/2}$ regularisation is suitable for this problem, both in terms of regularity and quality of the reconstruction.

Future research directions include the transition to 3D models and the generalisation to piecewise‐constant conductivities in the torso. In this case, it is still possible to leverage on the boundary integral formulation. From the mathematical perspective, a general result on the parametric regularity of the inverse problem is still missing. Such a result would help to shed some light on the sub‐optimal convergence rate observed in our experiments and the apparent lack of parametric regularity of the inverse problem with TV regularisation. Finally, from the clinical perspective, we shall further investigate how segmentation uncertainty may be modelled. As a matter of fact, the uncertainty likely results from multiple sources. Besides noise, CMR images are affected by breath holding, which may limit the volume of the heart and spatio‐temporal mis‐registration. The segmentation process may amplify the acquisition uncertainty, depending on the method. Importantly, the segmentation is sometimes a non‐smooth operation, in the sense that small perturbation in the images could yield large and possibly topological changes in the contour. Therefore, special care will be needed in order to correctly model the uncertainty from clinical data.

## Endnotes

^1^
For the collocation method, we require also $\chi \left(t,\omega \right)\in C^{2}\left(\bar{D_{\mathrm{ref}}}\right)$ for $t\in \left[0,T\right],\omega \in \Omega$.

^2^

https://www.blender.org

## Appendix A. Collocation method

Considering the parameterisations $\gamma_{\Gamma }:\left[0,1\right)\to \Gamma$ and $\gamma_{\sum }:\left[0,1\right)\to \sum$, the Dirichlet‐to‐Neumann map (4) in dimension d=2 reads, for $\Phi \in \left\{\Gamma ,\sum \right\}$ and $s\in \left[0,1\right)$,

$$-\frac{1}{4\pi }\sum_{\Psi \in \left\{\Gamma ,\sum \right\}}\int_{0}^{1}k_{\Phi ,\Psi }^{V}\left(s,r\right){\tilde{\rho }}_{1,\Psi }\left(r\right)d\;r=\frac{1}{2}\rho_{0,\Phi }\left(s\right)+\frac{1}{2\pi }\sum_{\Psi \in \left\{\Gamma ,\sum \right\}}\int_{0}^{1}k_{\Phi ,\Psi }^{K}\left(s,r\right)\rho_{0,\Psi }\left(r\right)d\;r,$$

where

$$k_{\Phi ,\Psi }^{V}\left(s,r\right)≔\log∥\gamma_{\Phi }\left(s\right)-\gamma_{\Psi }\left(r\right){∥}_{2}^{2},\;k_{\Phi ,\Psi }^{K}\left(s,r\right)≔\frac{{\langle \gamma_{\Phi }\left(s\right)-\gamma_{\Psi }\left(r\right),n_{r,\Psi }\rangle }_{2}}{∥\gamma_{\Phi }\left(s\right)-\gamma_{\Psi }\left(r\right){∥}_{2}^{2}}∥\gamma_{\Psi }^{\prime }\left(r\right){∥}_{2}$$

and

$$\rho_{0,\Phi }\left(s\right)≔y\left(\gamma_{\Phi }\left(s\right)\right),\;{\tilde{\rho }}_{1,\Phi }\left(s\right)≔\frac{\partial y}{\partial n_{s,\Phi }}\left(\gamma_{\Phi }\left(s\right)\right)∥\gamma_{\Phi }^{'}\left(s\right){∥}_{2}.$$

We consider the $n_{\Gamma }$‐points discretisation of Γ and the $n_{\sum }$‐points discretisation of ∑, where $n_{\Gamma }$ and $n_{\sum }$ are even numbers, and, for $\Phi \in \left\{\Gamma ,\sum \right\}$, we represent $\rho_{0,\Phi }$ and ${\tilde{\rho }}_{1,\Phi }$ as a linear combination of the trigonometric Lagrange polynomials $L_{j}$, for $j=0,\ldots ,n_{\Phi }-1$, that is,

$$\rho_{0,\Phi }\left(s\right)=\sum_{j=0}^{n_{\Phi }-1}\rho_{0,j}^{\Phi }L_{j}\left(s\right)\;\text{and}\;{\tilde{\rho }}_{1,\Phi }\left(s\right)=\sum_{j=0}^{n_{\Phi }-1}{\tilde{\rho }}_{1,j}^{\Phi }L_{j}\left(s\right).$$

Note that, considering the discretisation $s_{i}=i/n_{\Phi }$ for $i=0,\ldots ,n_{\Phi }-1$, it holds $L_{j}\left(s_{i}\right)=\delta_{i,j}$. Using the representation of $\rho_{0,\Phi }$ and ${\tilde{\rho }}_{1,\Phi }$, we end up with

$$\begin{array}{l} -\frac{1}{4\pi }\sum_{\Psi \in \left\{\Gamma ,\sum \right\}}\sum_{j=0}^{n_{\Psi }-1}\left(\int_{0}^{1}k_{\Phi ,\Psi }^{V}\left(s_{i},r\right)L_{j}\left(r\right)d\;r\right){\tilde{\rho }}_{1,j}^{\Psi }\\ =\frac{1}{2}\rho_{0,i}^{\Phi }+\frac{1}{2\pi }\sum_{\Psi \in \left\{\Gamma ,\sum \right\}}\sum_{j=0}^{n_{\Psi }-1}\left(\int_{0}^{1}k_{\Phi ,\Psi }^{K}\left(s_{i},r\right)L_{j}\left(r\right)d\;r\right)\rho_{0,j}^{\Psi } \end{array}$$

for $i=0,\ldots ,n_{\Phi }-1$ and $\Phi \in \left\{\Gamma ,\sum \right\}$. The goal is now to discretise the above equation. To do so, we consider the discretisation $r_{j}=j/n_{\Psi }$ for $j=0,\ldots ,n_{\Psi }-1$ and we distinguish between four different kinds of matrices.

In the case Ψ≠Φ, the kernel functions do not exhibit singularities. Hence, we may directly employ the trapezoidal rule to obtain the collocation approximation of the corresponding boundary integral operators. Using the trapezoidal rule, we have

$$-\frac{1}{4\pi }\sum_{j=0}^{n_{\Psi }-1}\left(\int_{0}^{1}k_{\Phi ,\Psi }^{V}\left(s_{i},r\right)L_{j}\left(r\right)d\;r\right){\tilde{\rho }}_{1,j}^{\Psi }\approx -\frac{1}{4\pi n_{\Psi }}\sum_{j=0}^{n_{\Psi }-1}k_{\Phi ,\Psi }^{V}\left(s_{i},r_{j}\right){\tilde{\rho }}_{1,j}^{\Psi },$$

therefore, we define the matrix $V_{\Phi \Psi }\in {\mathbb{R}}^{n_{\Phi }\times n_{\Psi }}$ whose entries, for $i=0,\ldots ,n_{\Phi }-1$ and $j=0,\ldots ,n_{\Psi }-1$, are

$${\left(V_{\Phi \Psi }\right)}_{i,j}≔-\frac{1}{4\pi n_{\Psi }}k_{\Phi ,\Psi }^{V}\left(s_{i},r_{j}\right).$$

Similarly, by the trapezoidal rule we have

$$\frac{1}{2\pi }\sum_{j=0}^{n_{\Psi }-1}\left(\int_{0}^{1}k_{\Phi ,\Psi }^{K}\left(s_{i},r\right)L_{j}\left(r\right)d\;r\right)\rho_{0,j}^{\Psi }\approx \frac{1}{2\pi n_{\Psi }}\sum_{j=0}^{n_{\Psi }-1}k_{\Phi ,\Psi }^{K}\left(s_{i},r_{j}\right)\rho_{0,j}^{\Psi }.$$

Hence, we define the matrix $K_{\Phi \Psi }\in {\mathbb{R}}^{n_{\Phi }\times n_{\Psi }}$ whose entries, for $i=0,\ldots ,n_{\Phi }-1$ and $j=0,\ldots ,n_{\Psi }-1$, are

$${\left(K_{\Phi \Psi }\right)}_{i,j}≔\frac{1}{2\pi n_{\Psi }}k_{\Phi ,\Psi }^{K}\left(s_{i},r_{j}\right).$$

If Ψ=Φ the kernel functions exhibit a singularity for s=r. This singularity can be dealt with as follows: We split

$$k_{\Phi ,\Phi }^{V}\left(s,r\right)=k_{\Phi ,\Phi }^{V,1}\left(s,r\right)+k_{\Phi ,\Phi }^{V,2}\left(s,r\right),$$

where

$$k_{\Phi ,\Phi }^{V,1}\left(s,r\right)≔\log\frac{∥\gamma_{\Phi }\left(s\right)-\gamma_{\Phi }\left(r\right){∥}_{2}^{2}}{4\sin^{2}\left(\pi \left(s-r\right)\right)}$$

and

$$k_{\Phi ,\Phi }^{V,2}\left(s,r\right)≔\log\left(4\sin^{2}\left(\pi \left(s-r\right)\right)\right).$$

Then, for the first term, using again the trapezoidal rule, we arrive at

$$-\frac{1}{4\pi }\sum_{j=0}^{n_{\Phi }-1}\left(\int_{0}^{1}k_{\Phi ,\Phi }^{V,1}\left(s_{i},r\right)L_{j}\left(r\right)d\;r\right){\tilde{\rho }}_{1,j}^{\Phi }\approx -\frac{1}{4\pi n_{\Phi }}\sum_{j=0}^{n_{\Phi }-1}k_{\Phi ,\Phi }^{V,1}\left(s_{i},r_{j}\right){\tilde{\rho }}_{1,j}^{\Phi }.$$

Since

$$\lim_{s\to r}k_{\Phi ,\Phi }^{V,1}\left(s,r\right)=\log{\left\|\frac{\gamma_{\Phi }^{'}\left(r\right)}{2\pi }\right\|}_{2}^{2},$$

we set

$$k_{\Phi ,\Phi }^{V,1}\left(s_{i},r_{j}\right)≔\log{\left\|\frac{\gamma_{\Phi }^{'}\left(r_{j}\right)}{2\pi }\right\|}_{2}^{2}$$

for $s_{i}=r_{j}$ and we define the matrix $V_{\Phi \Phi }^{1}\in {\mathbb{R}}^{n_{\Phi }\times n_{\Phi }}$ with

$${\left(V_{\Phi \Phi }^{1}\right)}_{i,j}≔-\frac{1}{4\pi n_{\Phi }}k_{\Phi ,\Phi }^{V,1}\left(s_{i},r_{j}\right)\;\text{for}\;i,j=0,\ldots ,n_{\Phi }-1.$$

Since $n_{\Phi }$ is an even number, we have $n_{\Phi }=2m_{\Phi }$ and for the second term it holds

$$-\frac{1}{4\pi }\sum_{j=0}^{n_{\Phi }-1}\left(\int_{0}^{1}k_{\Phi ,\Phi }^{V,2}\left(s_{i},r\right)L_{j}\left(r\right)d\;r\right){\tilde{\rho }}_{1,j}^{\Phi }\approx -\frac{1}{4\pi }\sum_{j=0}^{n_{\Phi }-1}R_{j}\left(s_{i}\right){\tilde{\rho }}_{1,j}^{\Phi },$$

where

$$R_{j}\left(s_{i}\right)=-\frac{1}{m_{\Phi }}\left(\sum_{k=1}^{m_{\Phi }-1}\frac{1}{k}\cos\left(2\mathrm{\pi k}\left(s_{i}-r_{j}\right)\right)+\frac{1}{n_{\Phi }}\cos\left(2\pi m_{\Phi }\left(s_{i}-r_{j}\right)\right)\right),$$

cp. Reference 12. This gives rise to the matrix $V_{\Phi \Phi }^{2}\in {\mathbb{R}}^{n_{\Phi }\times n_{\Phi }}$ with

$${\left(V_{\Phi \Phi }^{2}\right)}_{i,j}≔-\frac{1}{4\pi }R_{j}\left(s_{i}\right)\;\text{for}\;i,j=0,\ldots ,n_{\Phi }-1.$$

Finally, we set

$$V_{\Phi \Phi }≔V_{\Phi \Phi }^{1}+V_{\Phi \Phi }^{2}\in {\mathbb{R}}^{n_{\Phi }\times n_{\Phi }}.$$

For the double layer operator, we have

$$\frac{1}{2\pi }\sum_{j=0}^{n_{\Phi }-1}\left(\int_{0}^{1}k_{\Phi ,\Phi }^{K}\left(s_{i},r\right)L_{j}\left(r\right)d\;r\right)\rho_{0,j}^{\Phi }\approx \frac{1}{2\pi n_{\Phi }}\sum_{j=0}^{n_{\Phi }-1}k_{\Phi ,\Phi }^{K}\left(s_{i},r_{j}\right)\rho_{0,j}^{\Phi }.$$

Since

$$\lim_{s\to r}\;k_{\Phi ,\Phi }^{K}\left(s,r\right)=\frac{{\langle \gamma_{\Phi }^{''}\left(r\right),n_{r,\Phi }\rangle }_{2}}{2∥\gamma_{\Phi }^{'}\left(r\right){∥}_{2}},$$

we set

$$k_{\Phi ,\Phi }^{K}\left(s_{i},r_{j}\right)≔\frac{{\langle \gamma_{\Phi }^{''}\left(r_{j}\right),\;n_{r_{j},\Phi }\rangle }_{2}}{2∥\gamma_{\Phi }^{'}\left(r_{j}\right){∥}_{2}}$$

for $s_{i}=r_{j}$ and we define the matrix $K_{\Phi \Phi }\in {\mathbb{R}}^{n_{\Phi }\times n_{\Phi }}$ with

$${\left(K_{\Phi \Phi }\right)}_{i,j}≔\frac{1}{2\pi n_{\Phi }}k_{\Phi ,\Phi }^{K}\left(s_{i},r_{j}\right)\;\text{for}\;i,j=0,\ldots ,n_{\Phi }-1.$$

## Appendix B. Forward problem data

The evolution in time of the pericardial potential at the point $\gamma_{\sum \left(t,\omega \right)}\left(0\right)$ from which the stimulus is delivered is given, for $t\in \left[0,T\right)$, by

$$u\left(\gamma_{\sum \left(t,\omega \right)}\left(0\right),t\right)=u_{\mathrm{dep}}\left(t\right)+u_{\mathrm{rep}}\left(t\right),$$

with

$$u_{\mathrm{dep}}\left(t\right)=-25\frac{\tanh\left(2\left(\left(t/T-0.18\right)-\left\lfloor 0.5+\left(t/T-0.18\right)\right\rfloor \right)/0.1\right)}{\cosh{\left(2\left(\left(t/T-0.18\right)-\left\lfloor 0.5+\left(t/T-0.18\right)\right\rfloor \right)/0.1\right)}^{2}}$$

and

$$u_{\mathrm{rep}}\left(t\right)=\frac{25}{2\sqrt{2\pi }}\left(\exp\left(-100{\left(t/T-0.63\right)}^{2}\right)+\exp\left(-100{\left(t/T+0.37\right)}^{2}\right)\right).$$

For the other points on the pericardium $\sum \left(t,\omega \right)$, the potential is shifted in time according to the arrival time of the stimulus. For $s\in \left[0,1\right)$, the shift is given by

$$\delta \left(s\right)=0.22\left(\cos\left(2\mathrm{\pi s}-\pi \right)+1\right)/2$$

and the pericardial pericardial is given by

$$u\left(\gamma_{\sum \left(t,\omega \right)}\left(s\right),t\right)=u_{\mathrm{dep}}\left(t-\delta \left(s\right)T\right)+u_{\mathrm{rep}}\left(t-\delta \left(s\right)T\right).$$
